# How and when team-member exchange influences knowledge hiding behaviors: A moderated dual-pathway model

**DOI:** 10.1016/j.heliyon.2024.e28373

**Published:** 2024-03-22

**Authors:** Zijun Zhang, Yoshi Takahashi

**Affiliations:** Graduate School of Humanities and Social Sciences, Hiroshima University, Hiroshima, Japan

**Keywords:** Knowledge hiding, Team-member exchange, Job embeddedness, Work alienation, Learning goal orientation

## Abstract

**Purpose:**

This study explored the influence of team member exchange on employees’ knowledge hiding behaviors via job embeddedness and work alienation, with learning goal orientation acting as the boundary condition.

**Method:**

ology: This study adopted a quantitative multi-study research methodology to validate the proposed hypotheses, combining a time-lagged field study with 459 in-service employees and a scenario-based experiment with 128 university students at a northern university in China.

**Findings:**

In Study 1 (field study), team-member exchange was negatively associated with knowledge hiding, and job embeddedness and work alienation mediated this relationship. Perceptions of learning goal orientation can amplify the effect of team-member exchange on job embeddedness and work alienation, which in turn reduces knowledge hiding behaviors. A subsequent experiment (Study 2) almost replicated and supported these findings, but work alienation did not play a role as an intermediary in the relationship between team member exchange and knowledge hiding behavior.

**Practical implications:**

Managers should stimulate social exchanges among team members to inhibit knowledge hiding behaviors and prioritize individuals exhibiting higher learning goal orientations when deciding whom to hire.

**Originality:**

This research identifies and rationalizes how (underlying mechanisms) and when (contingencies) team-member exchange can make a difference in employees’ knowledge hiding behaviors, expanding and advancing further research on the knowledge hiding phenomenon from a team perspective.

## Highlights

• TMX was important for inhibiting employees' knowledge hiding behaviors.
• Job embeddedness mediates the relationship between TMX and knowledge hiding.
• LGO was imperative in amplifying the positive effects of TMX in the workplace.

## 1 Introduction

In the era of the knowledge economy, knowledge has been viewed as a priceless power to gain competitive advantages and ensure organizational survival in a fiercely competitive environment [1]. The capability to leverage and control valuable knowledge is important to obtain organizational efficiency and success [2]. Therefore, an increasing number of organizations have been spending considerable time and effort developing their own knowledge management systems to facilitate knowledge transfer and improve organizational performance [2]. However, people's willingness to share their knowledge is not facilitated as expected. Instead, numerous knowledge hiding cases have been reported at various institutions. A significant majority of employees in North America, namely 76%, tend to retain their knowledge and refuse to disseminate it to their co-workers to ensure their irreplaceability in their organizations [3]. In this regard, we can predict potential consequences if knowledge hiding behaviors go unchecked, such as reciprocal knowledge hiding [4] and poor team creativity [5]. Thus, there has been an increased recognition that knowledge hiding needs to be given more consideration for further study, such as what it is and how it comes into being. Exploring the constructs of knowledge hiding could raise people's alarm and resistance towards this counterproductive workplace behavior, which has important theoretical and practical implications for mitigating the instances of knowledge hiding in the workplace.

According to Connelly et al. [3], knowledge hiding is defined as an intentional attempt to conceal or withhold knowledge requested by others. The intentional nature and knowledge requests have been highlighted as the most distinctive characteristics that segregate knowledge hiding from other categories of knowledge-related behaviors, in particular, the concept of knowledge hiding does not represent a complete antithesis to knowledge sharing [3]. Knowledge sharing and knowledge hiding are driven by different motives [3]. While knowledge sharing mainly comes from the pro-social motive of sharing valuable knowledge by transferring it for to help others, knowledge hiding has been regarded as an unethical and deceptive behavior originating from antisocial or instrumental motivation by preventing co-workers’ knowledge acquisition through an intentional concealment of requested knowledge [3,4,6]. In this scenario, investigating the formation and development of knowledge hiding by equating it with a lack of knowledge sharing is difficult, even though approximately 50% of all papers published in the *Journal of Knowledge Management* in 2015 focused on rationalizing different facets of the knowledge-sharing phenomenon [4]. Therefore, inhibiting employees’ knowledge hiding behaviors requires separate and independent research on other similar knowledge-related constructs.

The discussion about “teamwork” and “workgroup” is prevalent in today's organizational behavior research. Reich [7] wrote, “if we are to compete effectively in today's world, we must begin to celebrate collective entrepreneurship, endeavors in which the whole of the effort is greater than the sum of individual contributions (p.78). We need to honor our teams more, our aggressive leaders and maverick geniuses less.” However, extant research emphasizes the link between knowledge hiders and seekers as a basis for constructing the nomological framework of knowledge hiding, resulting in a dyadic focus on this concept [3,6]. Thus, how and when individuals would engage in knowledge hiding behaviors within teams is still unclear. Therefore, based on the social information processing (SIP) theory [8], we argue that team member exchange (TMX), as a representative team-level construct, is an important antecedent of knowledge hiding, which is a microcosm of team-level social relations in the workplace. Team members could make a difference on individuals' attitudes, behaviors, and performance, as individuals could acquire informational cues from interpersonal interactions with co-workers, directly or indirectly, which could be used to create and interpret workplace affairs [8,9]. TMX, a collection of social interpersonal relations, characterized by mutual trust and respect, reminds employees of the importance of peer support and assistance [10]. Thus, TMX may inhibit knowledge hiding behaviors in team settings.

Along with the main relationship between TMX and knowledge hiding, we also aim to clarify the underlying mechanisms through which TMX exerts a negative influence on knowledge hiding. From an input-process-output perspective [11], it is necessary to highlight that process governed by organisms could effectively transfer interventions of input (TMX) to output (knowledge hiding), which are identified as intermediate mechanisms within this research. Empirical studies have already underlined the importance of and differentiation between cognitive [12] and behavioral processes [13] when analyzing influential pathways of forces that trigger or hinder people's engagement in knowledge hiding. Following Ma et al. [14], the incorporation of job embeddedness and work alienation as parallel mediators can significantly elucidate the internal and external mechanisms behind the relationship between TMX and knowledge hiding, especially in terms of why (cognitive) and how (behavioral) it comes into being. Furthermore, this research also considers when the effects of TMX on job embeddedness and work alienation are amplified or mitigated. This is because no organizational phenomenon can be caused by a single factor in isolation, and complex internal interactions between social environment and individual attributes should also be highlighted [15]. As instructed by Barron and Harackiewicz [16], learning goal orientation (LGO) generates a specific perceptual-cognitive framework, guiding individuals to process, appraise and react to achievement situations. Su [17] particularly suggests that reducing knowledge hiding requires not only maintaining long-standing social connections but also developing LGO to provide further resources and support. Accordingly, we suggest that LGO may moderate the way employees respond to external stimuli (TMX) by exerting a potential influence on employees' job embeddedness and work alienation, through which knowledge hiding would be further influenced.

**Fig. 1:**
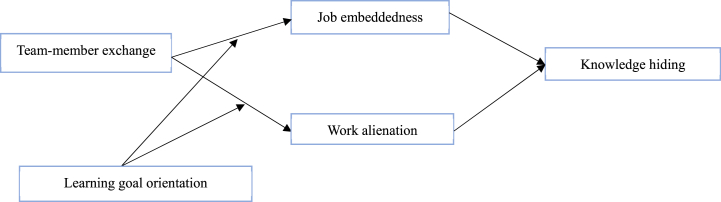
Research model.

In summary, our research makes several contributions by establishing a dual-pathway model to examine the relationship between TMX and knowledge hiding via job embeddedness and work alienation under the influence of one's LGO (see Fig. 1). First, this study sheds light on the importance of TMX and identifies it as an interfering antecedent of knowledge hiding. By highlighting the effect of TMX in reducing knowledge hiding, this research could inspire managers to effectively arrest knowledge hiding behavior in the workplace by developing harmonious relations through teamwork. Second, in response to Tan et al.’s [18] appeal, this study investigated explanatory mechanisms to rationalize the relationship between TMX and knowledge hiding through the lens of SIP by exploring dual mediating pathways: job embeddedness and work alienation. By doing so, this research also enriches the job embeddedness and work alienation literature by explaining employees' knowledge hiding behaviors. Finally, this study also provides insight into the interaction between TMX and LGO on the proposed mediators, which in turn minimizes the deleterious effects on employees' knowledge hiding behaviors.

## 2 Hypothesis development

### 2.1 Team-member exchange and knowledge hiding

TMX is a way of assessing reciprocal relationships between individuals and other team members. It mirrors an individual's comprehensive evaluations of the quality of work relationships within the team, including members providing help, ideas, and information to others and receiving recognition, feedback, and job resources from other members [19,20]. TMX is a collection of social relationships among team members who are identified as a group and assigned shared perceptions. Compared to the exchange imbalance caused by power and resource inequality between supervisors and subordinates, team members engaged in TMX processes are relatively independent and equal individuals aiming to strengthen internal reciprocity. Accordingly, high-quality TMX is generally characterized by mutual trust, respect, and coworker support [19], in which members value affiliative interpersonal relationships in team settings and are more willing to contribute to shared goal attainment and task completion. Thus, with the current flattening tendency of corporate structures, the within-group coworker relationship (TMX) is continuously emphasized to highlight its importance in stimulating employee effectiveness and improving organizational performance.

In the extant literature, TMX is widely regarded as a social-exchange construct analyzed from a relational perspective; however, the lens of social exchange is not very effective in explaining why and how TMX matters in individuals’ knowledge-related behaviors, as exchange ideology is not considered [21]. Not everyone would treasure social exchange and reciprocity with coworkers at the same level [21,22], indicating that the exclusion of personal values and norms would make the investigation of pairwise relationship between TMX and knowledge hiding incomplete. Further, Halbesleben et al. [23] also suggested that the social information derived from social environments reminded people what kinds of resources were more valuable and how to make full use of resources to gain competitive advantages and promote effectiveness; this refers to the lack of conservation of resources theory. In this manner, this study adopts the social information processing (SIP) theory as a theoretical foundation. SIP theory differs from other theories by emphasizing the importance of cognitive appraisal in interpreting social information acquired from social contexts, which in turn encourages people to adapt their judgments, choices, subsequent perceptions, and behaviors through such information processing procedures [8,24,25]. Moreover, Salancik and Pfeffer [8] stated that “one can learn most about individual behavior by studying the informational and social environment within which that behavior occurs and to which it adapts. (p.226)” Within this research, the lens of SIP could effectively rationalize the influence of TMX on knowledge hiding by elaborating the process through which coworkers could exert their influences.

Drawing on SIP theory, this study contributes to the understanding of individuals' engagement in knowledge hiding by focusing on cognitive processes under the influence of TMX. As instructed by Shetzer [26], emphasizing the process can help establish connections between diverse forms, multiple fundamental principles, and disciplinary viewpoints. Accordingly, this research provides a comprehensive picture by taking on a variety of mechanisms to rationalize the relationship between TMX and knowledge hiding. These procedures can be synthesized into three specific mechanisms: role-sending, attention-shifting, and role-modeling [8,27]. In line with the role-sending mechanism, individuals can analyze what is expected and desired through valuable information collected from other team members with shared perceptions and common goals, thus reducing role ambiguity and fulfilling their roles [28,29]. To rationalize the attention-shifting mechanism, social support from team members could be reconceptualized as a supportive strategy in team settings that could help individuals shift their attention from the negative to positive aspects of their work [30,31]. By continuously emphasizing the benefits gained from supportive interpersonal relationships, individuals seem to be more affectively committed to the organization and refuse to engage in knowledge hiding behaviors. Finally, higher-quality TMX could also encourage individuals’ vicarious learning behaviors from co-workers, characterized by mutual trust and respect through the role-modeling mechanism [28]. Thus, individuals would actively engage in social interactions and resource-sharing activities with other members, since they believe that their contributions could be acknowledged and valued by their workgroup; these people are less likely to conceal knowledge as a return to this supportive relationship. Based on these arguments, we hypothesize the following.

Hypothesis 1 TMX is negatively related to knowledge hiding.

### 2.2 The mediation of job embeddedness

Job embeddedness is defined as a broader collection of social, psychological, and financial factors that influence employees' attitudes towards retention in their current positions [32]. The concept of job embeddedness was first introduced by Mitchell et al. [32], and three main characteristics of job embeddedness were captured accordingly: link (formal and informal connections with leaders, co-workers, and institutions), fit (perceived compatibility within the organization or community) and sacrifice (the potential loss of material and psychological benefits when quitting) [32]. Job embeddedness is a significant predictor of a series of expected events, and also serves as a pivotal mediating framework connecting contextual factors and employees’ attitudes and behaviors [33,34]. From this viewpoint, job embeddedness provides new insights into the underlying mechanism by which specific workplace behaviors arise or disappear.

To our knowledge, in team settings, socially friendly co-workers could have a greater influence on employees' work attitudes and behaviors than their socially isolated counterparts. Drawing on the SIP theory, employees are capable of assimilating information from team members’ overt statements to form personal judgments and role expectations of their jobs under continuous role-sending processes in TMX conditions [8]. Thus, information asymmetry between organizations and employees will be largely improved via frequent social interactions among team members to reduce employee role ambiguity and clarify their job properties [27,28]; thus, they can better determine whether a specific job could provide the best personal fit. When personal characteristics fit well with the requirements of an organization, it is easier to persuade employees to stay. Moreover, the same relationship may be reasonable through a learning pathway [27]. The influence of a role model is associated with their position in social networks, and co-workers are more likely to be role models in teams [35]. In this regard, individuals can acquire information about what behavior is appreciated and what duty should be accepted as parts of their roles, shaping their perceptions of what they expect from their jobs, such as personal development and career growth. Employees tend to be more embedded in jobs with close interpersonal relationships and potential turnover costs.

Based on the extant literature, embedded employees are generally enmeshed with their jobs through strong social networks, higher person-job fit, and considerable sacrifices for their intention to quit [32], as they cannot afford the loss of benefits. With an increased desire for retention, employees will be internally motivated to perform the role preferred by the organization to meet or even exceed organizational expectations for their in-role performance. Embedded employees tend to show positive work behaviors and contribute more to the organization to maintain their jobs [36], so they are not willing to engage in knowledge hiding. Hence, such employees are opposed to withholding their expertise, skills, and abilities. Finally, researchers contended that being comfortably and safely attached to an entity provided a secure base from which individuals could be free of worries to explore something new and unknown [37], and that people were more likely to engage in exploratory or resource-enhancing activities when they perceived a higher level of job security and stability due to tight embeddedness in the organization. Thus, job embeddedness could hinder knowledgeable workers’ hidden intentions.

In summary, this study suggests that individuals who enjoy a higher level of TMX, which has been regarded as a key source of coworker support and informational cues about the workplace, are more likely to be embedded in their current jobs and avoid knowledge hiding behaviors. Therefore, we hypothesized the following.

Hypothesis 2 Job embeddedness mediates the relationship between TMX and knowledge hiding.

### 2.3 The mediation of work alienation

Fundamentally, work alienation is defined as a state of cognitive separation and psychological estrangement from work caused by working conditions that do not satisfy employees' self-fulfillment needs or expectations for career development [38]. A comprehensive definition of work alienation is still lacking, and mainstream research generally investigates workplace alienation from social [39] and psychological [40] perspectives. From the latter perspective, work alienation is conceptualized as being related to powerlessness, meaninglessness, social isolation, normlessness and self-estrangement [40]. The detrimental effects of work alienation have been highlighted in terms of job-related behaviors and performance [41], as alienation causes detachment and disillusionment. As many in-service employees suffer from a moderate level of work alienation, it is necessary to develop feasible strategies to mitigate the adverse effects of work alienation on employees’ work behaviors.

Negative aspects of work such as less autonomy, absence of variety, lack of interpersonal fulfillment, and increased levels of meaninglessness can define and predict employees' work alienation [42]. Based on the SIP theory, the social influence of TMX could also be exerted on employees by restructuring their attentional processes, such as reallocating personal attention to those who are more salient and favorable [8]. In this case, with a higher level of TMX, information derived from supportive interpersonal relations could regulate employees' negative affectivity by guiding their attention to the positive aspects of work or via motivating external stimuli [43,44]. Accordingly, employees could use this information to correct their negative perceptions of work; thus, they could be aware of the meaning and value of continuing to work as originally. Moreover, employees are less likely to develop avoidance orientation or withdrawal intention when they are aware of the recognition, support and assistance acquired from high-quality TMX relationships [10]. This attentional pathway seems to reduce individual's negative reactivity by transferring attention to a new and positive focus. Generally, TMX can reduce employees' feelings of work alienation by preventing them from being deeply affected by the negative aspects of their work.

Further, extant empirical research has already rationalized the underlying effect of work alienation on knowledge hiding. Guo et al. [45] illustrated that work alienation exhibited a positive correlation with knowledge hiding, driven by the intention to disengage from the loss spiral of job resources. Lee et al. [46] found a negative connection between work alienation and knowledge hiding under the influence of motivational climate. These findings are consistent with our assumption that work alienation is likely to be associated with destructive work outcomes. Therefore, based on the extant literature, work alienation generally leads to some withdrawal symptoms, such as the intention to quit and absenteeism [47]. Hence, employees experiencing higher levels of work alienation would feel disconnected and disappointed with their jobs and organizations. Thus, these people would be less interested in spending extra time and effort responding to their colleagues’ knowledge requests. In other words, work alienation hinders knowledge transfer and fosters hidden intentions and practices.

In short, as an information sharer, a team member could reduce the personal perception of work alienation by shifting one's attentional focus from negative to positive, which could inhibit employees' knowledge hiding behaviors in the workplace. Thus, we hypothesized the following.

Hypothesis 3 Work alienation mediates the relationship between TMX and knowledge hiding.

### 2.4 The moderation of learning goal orientation

LGO has been regarded as “a relatively stable dispositional trait that describes the extent to which individuals strive to understand something new or increase their level of competence in a given activity (p. 243)” [48]; it shapes and structures an individual's framework to interpret and respond to target behaviors or activities. LGO is associated with favorable work outcomes: for example, individuals with a strong orientation towards learning are primarily dedicated to achieving self-betterment and further development through the acquisition of new knowledge, skills, and abilities, and mastering new and challenging situations [49,50]. Given the salient importance of LGO, it has been considered a moderating factor with respect to job embeddedness and work alienation under the influence of TMX, as the goal orientation theory suggests that these orientations consist of people's experiences in specific situations, guiding their cognitive and emotional responses to situational affairs [51].

LGO may strengthen TMX's effect on employees' job embeddedness. From a social cognition perspective, individuals with LGO are intrinsically motivated to have greater expectations of further career success [48]. Thus, individuals with a higher LGO are more likely to cherish the existence of TMX, as various resources are required to fulfill their intensive needs to invest in different learning or training activities, which are very costly. Moreover, LGO impacts people's approaches to contextual feedback [52]. Learning-oriented individuals would value such kind of feedback and view it as an opportunity to learn and grow [53]. Accordingly, we can conclude that these people would function more favorably and effectively in TMX, which is in line with their advancement prospects, and gain what they need to achieve their goals. In this case, those with a higher LGO are more likely to interpret the resources and information acquired from TMX as support for fulfilling their learning needs. The potential sacrifice that employees experience once they leave their jobs is evident, which increases employees' willingness to be more embedded in their jobs [32]. Conversely, people with a lower LGO tend to be less motivated to value shared job resources; thus, they are less likely to gain ideal benefits from the TMX and gradually develop intentions to leave [54]. In this regard, we argue that LGO strengthens the positive relationship between TMX and job embeddedness.

Hypothesis 4 LGO moderates the relationship between TMX and job embeddedness, and this positive relationship is stronger when LGO is higher.

However, the potential impact of LGO as a moderator of the relationship between TMX and work alienation may vary significantly. Alienated employees often endure feelings of powerlessness and meaninglessness because their jobs do not meet employees’ needs for satisfaction and expectations [38,40]. As mentioned above, TMX possesses inherent value as a source of work-related knowledge, information, and skills, and LGO can strengthen the negative effect of TMX by fostering close communication and collaboration among team members [55]. By doing so, learning-oriented individuals take advantage of supportive TMX relations to meet their self-enhancement goals and generate more “mastery-oriented” responses [53]. In this case, individuals with LGO can better control the entire workflow and find more cognitive or psychological benefits that make them believe that their work is meaningful, which in turn encourages them to be less alienated from their work. Hence, leaning-oriented individuals enhance the efficiency of resource utilization and psychological involvement in the TMX process. Thus, we hypothesized the following.

Hypothesis 5 LGO moderates the relationship between TMX and work alienation, and this negative relationship is stronger when LGO is higher.

## 3 Overview of the research

This research is a combination of field and experimental study. In Study 1, we conducted a field study to explore the hypothesized model concerning the relationship between TMX and knowledge hiding using observational data from in-service employees in China. Observational research provides opportunities to achieve higher external validity concerning the generalizability and transportability of empirical findings [56]. Although valuable, observational research also presents certain limitations in terms of internal validity, as the existence of unmeasured confounders might compromise the accuracy of the results [56]. As a complementary analysis to Study 1, Study 2 conducted a scenario-based experiment utilizing samples of university students, owing to their convenience and accessibility [57]. Study 2 enhances the internal validity of this research and contributes to estimate the causality between TMX and knowledge hiding via job embeddedness and work alienation [58]. To conclude, both the field study and experimental study strengthen the robustness and validity of our research findings.

## 4 Study 1: field study

### 4.1 Sample and design

To reduce common method bias, we used a time-lagged research design for Study 1 with a two-week interval in China. The decision to select a two-week time lag was based on the recommendation of Dormann and Griffin [59] who advocate for the adoption of shortitudinal research designs instead of time intervals calculated by months. Considering their availability, we collected data through a professional platform Credamo and invited 600 in-service employees to participate in our research from February to March 2023. All respondents were required to fulfill the following two requirements: they were at least 22 years old, a common age to complete higher education in China; they were currently employed for no less than 40 h per week, that is, employed full-time. To ensurethe credibility of the research projects, data cleansing was adopted to remove invalid and missing data, such as inconsistent data or abnormal values. Furthermore, this study excluded respondents who did not provide accurate responses to the attention checks.

Before the investigation, we required all respondents to read the informed consent form and understand the relevant information for this kind of research to ensure that they voluntarily participated in the data collection process in an informed manner. All data were strictly confidential and used only for academic research. Accordingly, we distributed 600 questionnaires to the respondents at Time 1 and received 600 responses back. All participants were required to provide their demographic information, and report on the TMX and LGO scales. Two weeks later, 485 participants responded to the second survey. Participants who provided valid responses were invited to report their perceptions of job embeddedness, work alienation, and knowledge hiding at Time 2. Finally, 459 employees completed the survey at the two time points (a valid response rate of 76.5%). Regarding demographic information, 239 participants were female (52.1%), the average age of the sample was 33.38 years. The majority of participants held a bachelor's degree and above, which was 92.4%. The average duration of employment for the employees in our sample was 9.61 years at their current place of work.

To ensure the validity of our data, Study 1 involved a non-response bias test. Following the recommendations of Armstrong and Overton [60], we chose the “selective extrapolation” method to test the non-response bias, where the measures of both control and key research constructs for the respondents from the first 25% and those of the final 25% were tested through the Levene's test of homogeneity of variance and independent-samples *t*-test (see in Appendix A). The results revealed no significant difference between early and late respondents. Thus, we concluded that the non-response bias was not a serious issue in this sample, and the useable sample was not affected by non-response bias, that is, both early and late respondents of this study effectively represented the sample target population.

### 4.2 Measurements

The Chinese versions of all measures were developed using the widely employed translation-back translation methodology [61]. The participants were asked to respond to all items on a five-point Likert-type scale ranging from 1 = strongly disagree to 5 = strongly agree. We used the same measurement instructions as in Study 2, specifically:

LGO was measured using a five-item scale created by VandeWalle [50], which focused specifically on work settings rather than educational or sports settings. The participants indicated the extent to which they perceived LGO. Sample items included, “I am willing to select a challenging work assignment that I can learn a lot from; I often look for opportunities to develop new skills and knowledge.”

The 10-item TMX Quality Scale developed and improved by Seers et al. [20] was used to measure team members’ perceptions of the reciprocal exchange relationship between themselves and team members. The scale included the following: “I often make suggestions about better work methods to other team members; Others let me know when I affect their work; I am flexible about switching job responsibilities to make things easier for other members.”

Seven items were used to measure job embeddedness, using the scale developed by Crossley et al. [62]. Items such as, “I feel embeddedness in this organization” and “I simply could not leave this company that I work for” were included.

Work alienation was evaluated using an eight-item scale developed by Nair and Vohra [42]. Representative items included: “I actually don't enjoy work in this firm”; “I just put in my time to get paid”; “I have gradually become disillusioned about my work after joining this firm”; and “I do not feel like putting my best effort at work”.

Knowledge hiding was assessed using a 12-item scale adapted from Connelly et al. [3] to measure the likelihood of participants hiding knowledge from co-workers. It included items such as, “I would say I am not knowledgeable about the topic,” “I would agree to help him/her but never really intend to do so,” and “I would explain that the information is confidential and only available to people in the project team.”

Regarding control variables, the extant literature suggests that demographic variables such as age, gender, working tenure, and education might be relevant to knowledge hiding [3,63]. Hence, it was necessary to introduce different demographic variables in Study 1, including gender, age, tenure, and education. In this study, gender (0 = male; 1 = female) and education (0 = junior college; 1 = Bachelor's; 2 = Master's; 3 = PHD) were coded as dummy variables, and age and working tenure were evaluated by year.

### 4.3 Results

#### 4.3.1 Common method bias

Harman's single-factor test was employed in order to assess the potential presence of common method bias and its threat to our results of data analysis. All items were entered together into the exploratory factor analysis (EFA), revealing that the first principal component explained 36.56% of the total variance among the five characteristic roots greater than 1, far below the threshold of 50% for common method variance [64]. Accordingly, no solitary factor could explain the majority of the variance, indicating that the common method bias was not a significant concern in this study.

#### 4.3.2 Measurement model

Before measuring the whole conceptual framework, we conducted a series of confirmatory factor analyses using AMOS.26, to assess the model fitness and factor structure of all study variables (TMX, LGO, job embeddedness, work alienation and knowledge hiding). Table 1 shows the fitting indices of the five-factor model, indicating that the hypothesized model had a satisfactory fit ($\chi^{2}$/df = 1.436, RMSEA = 0.031, CFI = 0.979, TLI = 0.978, and SRMR = 0.035) and was better than all the alternative models.

**Table 1:** Confirmatory factor analysis.

	CMIN	DF	CMIN/DF	RMSEA	CFI	TLI	SRMR
Five factor model: hypothesized model	1161.458	809	1.436	0.031	0.979	0.978	0.035
Four factor model: TMX + LGO; JE; WA; KH;	2326.204	813	2.861	0.064	0.911	0.906	0.065
Three factor model: TMX + LGO; JE + WA; KH;	4714.522	816	5.778	0.102	0.770	0.758	0.098
Two factor model: TMX + LGO; JE + WA + KH;	7699.204	818	9.412	0.136	0.595	0.574	0.149
One factor model: TMX + LGO + JE + WA + KH;	10856.953	819	13.256	0.164	0.409	0.379	0.169

Notes: TMX = team member exchange; LGO = learning goal orientation; JE = job embeddedness; WA = work alienation; KH = knowledge hiding; RMSEA = Root-Mean-Square Root of Approximation; CFI=Comparative Fit Index; TLI = Tucker-Lewis Index; SRMR=Standardized Root Mean Squared Residual.

This study also examined the validity and reliability of the data. Table 2 shows the factor loadings, composite reliability (CR), average variance extracted (AVE), and Cronbach's Alpha. The Cronbach's Alpha of all measures ranged from 0.910 to 0.958, greater than the cutoff value of 0.7, indicating good reliability of these scales. In addition, the convergent validity of the measurement model was examined through standardized factor loadings, AVE, and CR. The factor loadings were not less than 0.50 and ranged from 0.754 to 0.914. Values for AVE ranged from 0.665 to 0.749, and CR ranged from 0.911 to 0.960, which were above the threshold values of 0.5 and 0.7 respectively, confirming good convergent validity of the proposed conceptual framework. Following this, discriminant validity was investigated using the criteria suggested by Fornell and Larcker [65] and Henseler et al. [66]. Table 3 shows that the square root values of AVE (diagonal amount) were greater than the corresponding intercorrelations among each variable (Fornell-Larcker criterion), and Table 4 indicates that the values for heterotrait-monotrait ratio of Correlations (HTMT) ranged between 0.245 and 0.501, far below the threshold value of 0.85. These results confirmed that the measurement model has an acceptable level of discriminant validity.

**Table 2:** Validity and reliability.

Variable	Item	Estimate	CR	AVE	Cronbach's Alpha
LGO	LGO1	0.775	0.911	0.673	0.910
	LGO2	0.850			
	LGO3	0.730			
	LGO4	0.850			
	LGO5	0.888			
JE	JE1	0.875	0.954	0.749	0.952
	JE2	0.819			
	JE3	0.853			
	JE4	0.911			
	JE5	0.849			
	JE6	0.835			
	JE7	0.914			
WA	WA1	0.844	0.947	0.693	0.946
	WA2	0.792			
	WA3	0.832			
	WA4	0.813			
	WA5	0.826			
	WA6	0.868			
	WA7	0.850			
	WA8	0.833			
KH	KH1	0.760	0.960	0.665	0.958
	KH2	0.817			
	KH3	0.883			
	KH4	0.810			
	KH5	0.792			
	KH6	0.869			
	KH7	0.824			
	KH8	0.771			
	KH9	0.875			
	KH10	0.830			
	KH11	0.754			
	KH12	0.784			
TMX	TMX1	0.828	0.958	0.698	0.958
	TMX2	0.820			
	TMX3	0.827			
	TMX4	0.802			
	TMX5	0.849			
	TMX6	0.862			
	TMX7	0.863			
	TMX8	0.834			
	TMX9	0.834			
	TMX10	0.831			

Notes: LGO: learning goal orientation; JE: job embeddedness; WA: work alienation; KH: knowledge hiding; TMX: team-member exchange; CR: composite reliability; AVE: average variance extracted.

**Table 3:** Fornell-Larcker criterion.

	TMX	JE	WA	KH	LGO
TMX	**0.835**				
JE	0.384	**0.866**			
WA	−0.343	−0.487	**0.832**		
KH	−0.387	−0.38	0.385	**0.815**	
LGO	0.496	0.247	−0.273	−0.347	**0.821**

Notes: TMX: team-member exchange; JE: job embeddedness; WA: work alienation; KH: knowledge hiding; LGO: learning goal orientation; The square roots of average variance extracted are in bold.

**Table 4:** HTMT criterion.

	TMX	JE	WA	KH	LGO
TMX					
JE	0.382				
WA	0.346	0.489			
KH	0.389	0.379	0.385		
LGO	0.501	0.245	0.254	0.35	

Notes: TMX: team-member exchange; JE: job embeddedness; WA: work alienation; KH: knowledge hiding; LGO: learning goal orientation.

#### 4.3.3 Descriptive statistics

Table 5 shows the means, standard deviations, and correlations for all the variables. The results showed that TMX was positively related to job embeddedness (r = 0.364, p < 0.01), but negatively related to work alienation (r = −0.330, p < 0.01) and knowledge hiding (r = −0.373, p < 0.01). Similarly, we found that knowledge hiding was negatively related to job embeddedness (r = −0.362, p < 0.01), but positively related to work alienation (r = 0.365, p < 0.01). In line with our expectations, the correlations between the main variables were supported.

**Table 5:** Means, standard deviations, and correlations.

	1	2	3	4	5	6	7	8	9
1.Gender									
2.Age	−0.004								
3.Tenure	−0.026	0.953**							
4.Education	0.221**	−0.027	−0.113*						
5.TMX	0.005	−0.056	−0.092*	0.069					
6.JE	−0.094*	0.051	0.040	0.072	0.364**				
7.WA	0.043	−0.043	−0.005	−0.060	−0.330**	−0.461**			
8.KH	0.034	−0.015	−0.002	−0.080	−0.373**	−0.362**	0.365**		
9.LGO	−0.072	0.001	−0.043	0.067	0.469**	0.226**	−0.239**	−0.328**	
Means	0.52	33.38	9.61	1.14	3.55	3.77	2.24	2.30	3.55
SD	0.50	7.99	7.06	0.55	1.06	0.95	0.96	0.97	0.85

Notes: TMX: team-member exchange; JE: job embeddedness; WA: work alienation; KH: knowledge hiding; LGO: learning goal orientation; SD: standard deviation; P*<0.5; p**<0.01.

#### 4.3.4 Hypothesis testing

Hierarchical linear regression was conducted to examine the proposed model via SPSS.24, and the results are presented in Table 6.

**Table 6:** Hierarchical linear regression.

	KH				JE			WA		
	M1	M2	M3	M4	M5	M6	M7	M8	M9	M10
Gender	0.055	0.051	0.020	0.006	−0.116*	−0.111*	−0.107*	0.060	0.056	0.049
	(1.151)	(1.141)	(0.468)	(0.149)	(-2.427)	(-2.511)	(-2.409)	(1.259)	(1.246)	(1.084)
Age	−0.078	0.039	0.093	0.141	0.083	−0.032	−0.037	−0.393*	${-0.293}^{+}$	${-0.279}^{+}$
	(-0.489)	(0.262)	(0.655)	(0.998)	(0.527)	(-0.213)	(-0.247)	(-2.484)	(-1.941)	(-1.855)
Tenure	0.064	−0.080	−0.110	−0.155	−0.032	0.110	0.122	0.367*	0.242	0.222
	(0.401)	(-0.534)	(-0.769)	(-0.109)	(-0.199)	(0.739)	(0.816)	(2.301)	(1.593)	(1.467)
Edu	-$0.087^{+}$	−0.074	−0.053	−0.049	$0.096^{+}$	$0.083^{+}$	$0.081^{+}$	−0.042	−0.031	−0.027
	(-1.747)	(-1.590)	(-1.198)	(-1.109)	(1.945)	(1.803)	(1.757)	(-0.850)	(-0.651)	(-0.579)
TMX		−0.373**	−0.244**	−0.170**		0.368**	0.351**		−0.322**	−0.292**
		(-8.515)	(-5.340)	(-3.416)		(8.417)	(7.156)		(-7.251)	(-5.849)
JE			−0.174**	−0.172**						
			(-3.586)	(-3.566)						
WA			0.204**	0.194**						
LGO			(4.271)	−0.157**			$0.103^{+}$			−0.138**
				(-3.136)			(1.967)			(-2.612)
INT				0.023			0.117*			−0.125*
				(0.503)			(2.470)			(-2.579)
$R^{2}$	0.010	0.147	0.232	0.253	0.021	0.153	0.167	0.020	0.122	0.141
Adjusted $R^{2}$	0.001	0.137	0.220	0.238	0.012	0.144	0.154	0.012	0.112	0.128
F	1.138	15.554**	19.478**	16.929**	2.412*	16.397**	12.934**	2.354	12.611**	10.607**

Notes: Edu: education; TMX: team-member exchange; JE: job embeddedness; WA: work alienation; LGO: learning goal orientation; INT: interaction; The value in parenthesis represents t-value for standardized coefficients. **p < 0.01; *p < 0.05; +p < 0.1.

**Main effect:**Hypothesis 1 posited a negative connection between TMX and knowledge hiding. After controlling for demographic factors including age, gender, working tenure, and education (see Model 1 in Table 6), there was a negative relationship between TMX and employees’ knowledge hiding behaviors (β = −0.373, p < 0.01). Thus, Hypothesis 1 was supported.

**Mediation effects:** Within the dual-pathway research model, we set TMX as the antecedent variable, job embeddedness and work alienation as mediators, and knowledge hiding as the dependent variable. As shown in Table 6, there was a positive correlation between TMX and job embeddedness (β = 0.368, p < 0.01; see Model 6 in Table 6), while job embeddedness exhibited a negative association with knowledge hiding (β = −0.174, p < 0.01; see in Model 3 in Table 6). The indirect effect of job embeddedness was also significant (indirect effect = −0.059, ${CI}_{95\%}$ = [−0.094，-0.024] excluding zero; see Table 7) through bootstrapping mediation analysis with 5000 replications as recommended by Hayes [67]. Additionally, TMX was negatively related to work alienation (β = −0.322, p < 0.01; see Model 9 in Table 6), and work alienation was positively related to knowledge hiding (β = 0.204, p < 0.01; see Model 3 in Table 6). The indirect effect of work alienation was also significant, as our expected (indirect effect = −0.06, ${CI}_{95\%}$ = [−0.098, −0.028] excluding zero; see Table 7). Thus, Hypotheses 2 and 3 were supported.

**Table 7:** Indirect effect.

Model	Indirect effect	SE	LLCI	ULCI
TMX-JE-KH	−0.059	0.0179	−0.094	−0.024
TMX-WA-KH	−0.060	0.0182	−0.098	−0.028

Notes: TMX: team-member exchange; JE: job embeddedness; WA: work alienation; KH: knowledge hiding; LLCI: lower limit confidence interval; ULCI: upper limit confidence interval.

**Fig. 2:**
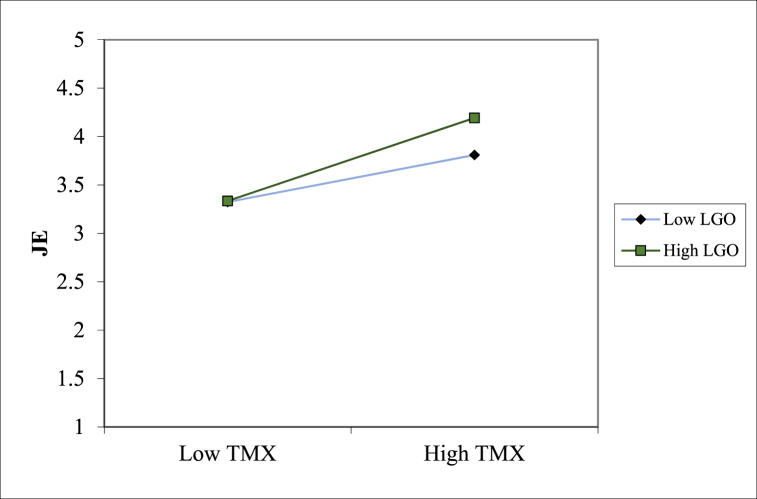
LGO as the moderator between TMX and job embeddedness.

**Moderation effect:**Table 6 also shows that the interaction of TMX and LGO yielded a positive impact on job embeddedness (β = 0.117, p < 0.05). To better interpret the moderation effect, we followed the protocol suggested by Preacher et al. [68] to plot the moderation at two levels of LGO (one standard deviation above and below the mean value of LGO). As shown in Fig. 2, the simple slope analysis indicated that the positive connection between TMX and job embeddedness was more pronounced for higher learning-goal-oriented employees (β = 0.406, p < 0.01) than for lower learning-goal-oriented employees (β = 0.229, p < 0.01).

**Fig. 3:**
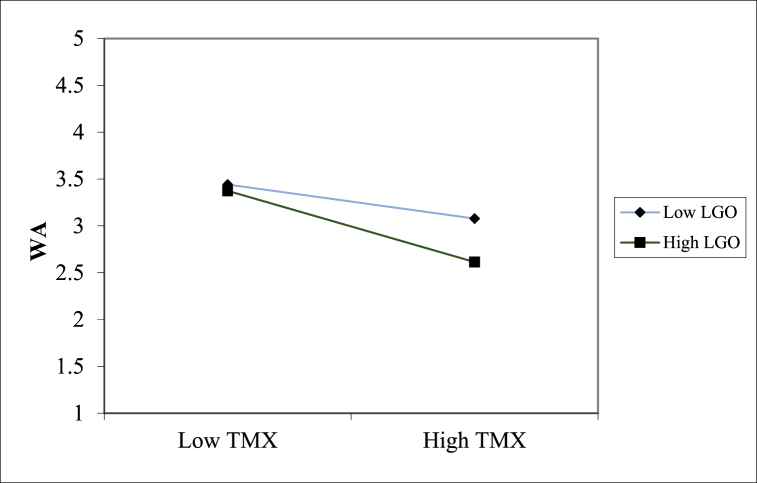
LGO as the moderator between TMX and work alienation.

Likewise, the interaction between TMX and LGO was also significant for work alienation (β = −0.125, p < 0.05). Using a similar simple slope analysis, we found that the negative relationship between TMX and work alienation was stronger for higher learning-goal-oriented employees (β = −0.360, p < 0.01) than for lower learning-goal-oriented employees (β = −0.171, p < 0.01), as shown in Fig. 3. TMX strongly fostered job embeddedness and reduced work alienation when people were characterized by higher levels of LGO. Thus, Hypotheses 4 and 5 were supported

## 5 Study 2 scenario-based experiment

### 5.1 Sample and design

The study population consisted of 128 undergraduate and postgraduate students who were enrolled in a university forum, out of 487 university students located in China (a valid response rate of 26.3%). The ages of all respondents in the study spanned from 18 to 28 years, with a calculated mean age of 21.59 years (SD = 1.80). Of all the students, 56.3% were female, and 42.6% had some working experience. In Study 2, participants were instructed to provide their responses to the proposed hypothetical scenario of the main variable. All 128 participants were randomly assigned to two conditions: high TMX and low TMX. We asked participants to report their feelings and corresponding behavioral intentions honestly in different scenarios. By measuring the participants' perceptions of LGO, we categorized the participants into two groups with low and high values of LGO based on the mean split approach. Accordingly, this experiment employed a two-by-two between-subjects research design (two conditions of TMX: low/high; two conditions (high/low) of LGO based on participants’ ratings about this construct). Similar to the Study 1, the results of the non-response bias test revealed that no significant differences were found between early and late respondents, indicating that students included in this experiment effectively represented the target population (see in Appendix B).

### 5.2 Manipulations

In both TMX conditions (high/low), participants were instructed to read a brief description of the hypothetical organization that they were currently working for and the job duties that they had to take on before beginning the experiment.

Please imagine being a sales agent in a famous security firm in this industry, which is of great importance to the local economic development and employment. You are now responsible for some work tasks, such as identifying potential clients, selling securities and commodities in investment and trading firms, and developing and implementing financial plans for individuals, businesses and organizations.

**TMX manipulation.** To manipulate TMX, this study described either a high or low social exchange relationship among teammates in the workplace. Previous research has successfully manipulated team member relationships through scenarios or vignettes [69].

(For the higher TMX condition) In your workplace, you like your teammates and enjoy the time working with them. Your teammates are helpful and supportive, giving you enough advice about what you need to do and how to be efficient in achieving work tasks. You find it easier to gain additional job resources from team members such as recognition, idea sharing, performance feedback, and latest news about market changes. You and your teammates recognize and celebrate each other’s unique strengths in the workplace. You are also more willing to do more than what is required of your job duties to help your teammates for their favors.

(For the lower TMX condition) In your workplace, you do not like your teammates and do not enjoy the time working with them. Your teammates are unhelpful and unsupportive by avoiding providing necessary information about what you need to do and how to be efficient in your daily work, even though you are working as a team. You find it difficult to gain additional job resources from team members such as recognition, idea sharing, performance feedback, and latest news about market changes. You and your teammates do not recognize and celebrate each other’s unique strengths in the workplace. You are less motivated to do more than what is required of your job duties as revenge against your teammates.

After the manipulation, participants were required to complete the 10-item TMX scale developed by Seers [20] (α = 0.98), and the response to this scale served as a manipulation check for TMX. Participants were also required to evaluate LGO (α = 0.79) using VandeWalle's [50] five-item scale, job embeddedness (α = 0.97) using Crossley et al.’s [62] seven-item scale, work alienation (α = 0.96) using Nair and Vohra's [42] scale and knowledge hiding (α = 0.98) using Connelly et al.’s [3] scale.

### 5.3 Results

**Manipulation check:** Prior to testing the proposed hypotheses, we adopted a manipulation check for TMX to confirm the extent to which participants agreed with the TMX statement. The results of an independent-samples *t-test* unveiled a noteworthy difference between the two conditions of TMX (t (128) = −3.06, p = 0.03), thus supporting the successful manipulation of TMX.

**Fig. 4:**
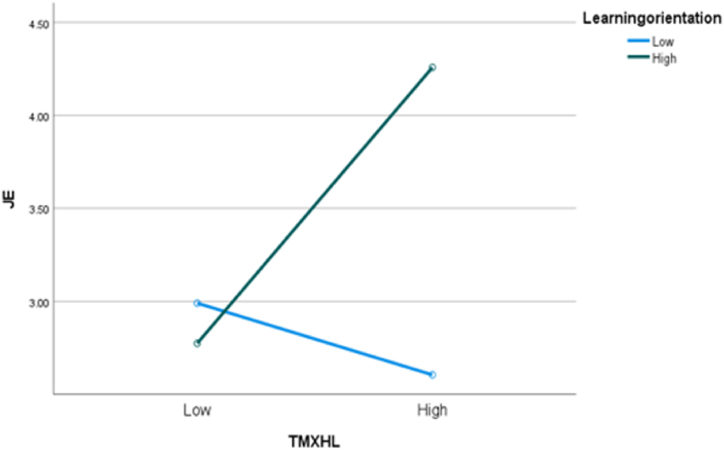
The moderating role of LGO between TMX and knowledge hiding.

**Fig. 5:**
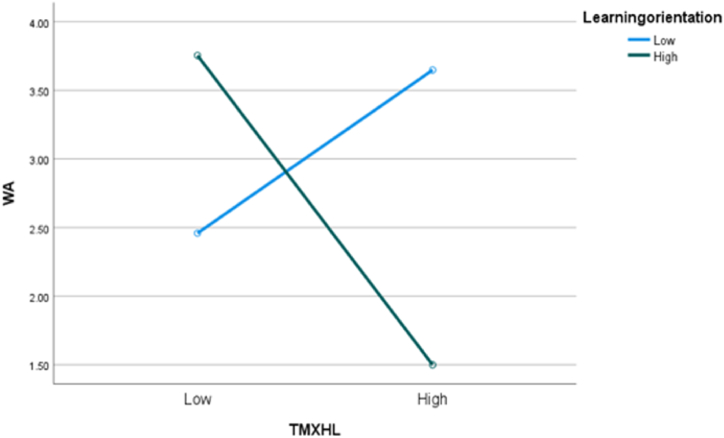
The moderating role of LGO between TMX and knowledge hiding.

**Main effect and moderation effect:** Turning to knowledge hiding as the dependent variable, the analysis of variance (ANOVA) indicated that there was a significantly negative association between TMX and knowledge hiding (F (1, 126) = 4.58, p < 0.05; M_high_ = 2.57, SD = 1.23; M_low_ = 3.05, SD = 1.31), thus supporting Hypothesis 1. We then conducted another two-way ANOVA to separately test whether TMX was associated with job embeddedness and work alienation under the influence of LGO. The mean score for job embeddedness in the high TMX scenario was significantly higher than that in the low TMX scenario (M_high_ = 3.41, SD = 1.23; M_low_ = 2.88, SD = 1.25; F (1, 124) = 7.98, p < 0.05). The mean score for work alienation in the higher TMX scenario was significantly lower than that in the low TMX scenario (M_high_ = 2.61, SD = 1.33; M_low_ = 3.13, SD = 1.15; F (1, 124) = 11.83, p < 0.05). Furthermore, the interaction effects of TMX and LGO on job embeddedness and work alienation were significant as shown in Fig. 4, Fig. 5(F (1, 124) = 23.08, p < 0.05; F (1, 124) = 123.403, p < 0.05), indicating that Hypotheses 4 and 5 were also supported in Study 2.

**Causal mediation analysis:** Considering the importance of internal causal inferences of the proposed mediated pathways, the sequential ignorability assumption must be satisfied [70]. Thus, Study 2 adopted causal mediation analysis to investigate the mediating influences of job embeddedness and work alienation, to ensure that there were no unmeasured confounders producing biased results. As expected, the mediating effect of job embeddedness was still significant (−0.195*) in Study 2, whereas the mediating effect of work alienation was not replicated (−0.069) in the way we expected. In this case, we concluded that Hypothesis 2 was supported, but Hypothesis 3 was not (see Table 8).

**Table 8:** Causal mediation analysis.

Variables	ACME	ADE	Total Effect	Value of ρ at which ACME = 0
TMX-JE-KH	−0.195* (−0.401, −0.040)	−0.272 (−0.674,0.150)	−0.467*(-0.885, −0.030)	−0.4
TMX-WA-KH	−0.069 (−0.209, 0.020)	−0.401 (−0.826,0.040)	−0.469* (−0.896, −0.050)	**/**

Notes: TMX: team-member exchange; JE: job embeddedness; WA: work alienation; KH: knowledge hiding; ACME = Average casual mediation effect; ADE = Average direct effect; **p < 0.01; *p,0.05.

**Sensitivity analysis:** The causal mediation estimates would continuously be identified only if the sequential ignorability assumption held true. Sequential ignorability assumptions indicate that there are no unmeasured confounders or covariates for the treatment-mediator, mediator-outcome, or treatment-outcome relationships [71]. However, there might exist some potential unobserved post-treatment confounders that could violate the relationship between the mediator and outcome, as the mediators were not randomized in our experiment [70]. Accordingly, it is necessary to conduct a sensitivity analysis to promote the robustness of the causal mediation estimates by determining the extent to these estimates might be confounded by observed or unobserved covariates.

**Fig. 6:**
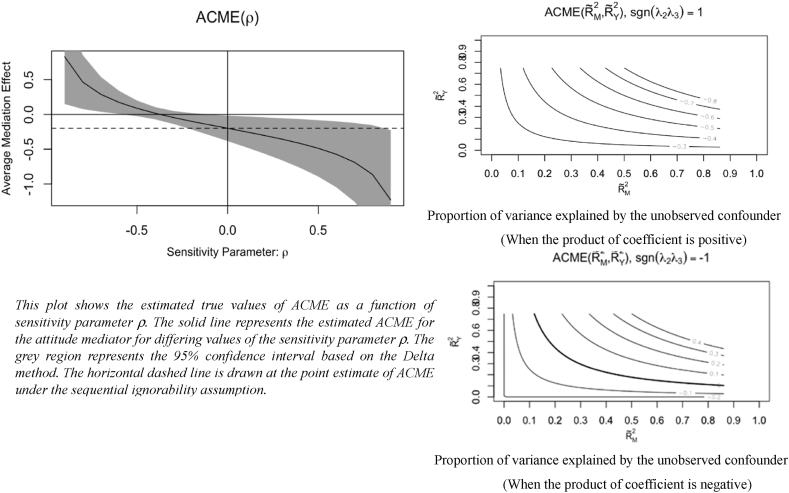
Sensitivity analysis.

Fig. 6 shows the results of the sensitivity analysis in accordance with the sensitivity parameter ρ, which is the residual correlation between the error items in the relationship between the mediator and outcome [70]. The results of the sensitivity analysis indicated that the sign of the Average Causal Mediation Effect (ACME) under the sequential ignorability assumption did not change until ρ was less than −0.4, representing a commonly accepted ρ value for moderate robustness in the field of social science [70,72]. The confidence interval for the ACME contained the value of zero only when −0.5< ρ < −0.2 even though we took the sampling variability into consideration [70]. Alternatively, this study also explained the same sensitivity analysis through the interpretation of the determination of coefficients to facilitate easier understanding [70]. Fig. 6 also shows the contours for $\tilde{R_{M}^{2}}$ and $\tilde{R_{Y}^{2}}$ which reveal the proportion of variance elucidated by the unobserved covariates [73]. Thus, if the product of the coefficients for unobserved confounders was positive, the original finding for the direction of ACME was perfectly robust to the violation of the sequential ignorability assumption, as the causal estimate of job embeddedness would always be negative at any value of $\tilde{R_{M}^{2}}$ and $\tilde{R_{Y}^{2}}$. However, when the product of the coefficients for the unobserved confounders was negative, the original finding was less robust than the first one. The original findings were nullified; the sign of the mediation estimate (job embeddedness) became positive when the unobserved confounder explained at least 40% of the variance in job embeddedness and 30% of the variance in knowledge hiding, which could be viewed as a relatively high proportion that is difficult to achieve. In summary, these results confirmed the moderate robustness of the mediation effect of job embeddedness in the relationship between TMX and knowledge hiding. Additionally, it was not necessary to run a sensitivity analysis for the casual mediation effect of work alienation, as ACME was not significant.

## 6 General discussion

Consistent with our hypotheses, the results of Study 1 indicated that TMX could effectively hinder the emergence of knowledge hiding behaviors in the workplace. Under the influence of TMX, which is characterized by mutual trust and coworker support, observations of social relations and peer interactions can help individuals acquire important information that reminds them of appreciated and encouraged attitudes and behaviors within a workgroup [8], including avoiding knowledge hiding behaviors. This result is consistent with the findings of Jah and Varkkey [74], who found that knowledge hiding was low for contexts in which there was a positive reciprocal relationship with co-workers. Study 1 also confirmed that job embeddedness and work alienation were important mediators through which the benefits of TMX could be converted into employee knowledge-related behaviors. Finally, although we determined how knowledge hiding was shaped by TMX, it was also necessary to explore the conditions under which the magnitude of these effects would be amplified or mitigated. In Study 1, TMX and LGO interacted to enhance job embeddedness and reduce work alienation, indicating that this research not only emphasizes the importance of team concept (TMX) but also of self-disposition (LGO). Specifically, the relationship between TMX and job embeddedness is moderated by individuals’ perceptions of LGO, whereby the connection is more pronounced at a heightened level of LGO. Similarly, LGO could amplify the negative influence of TMX on work alienation, which, in turn, reduces the emergence of knowledge hiding behaviors.

To increase the internal validity of our findings, Study 2 retested all the proposed hypotheses in a conceptual framework via a scenario-based experiment, as the experimental design offered the highest level of control and reduced the potential bias of unobserved covariates. Most of the results were replicated, except for the mediating effect of work alienation in the relationship between TMX and knowledge hiding. However, the mediation effect of job embeddedness was supported under the sequential ignorability assumption through a causal mediation analysis, and a moderate degree of robustness was demonstrated via a sensitivity analysis at the same time. Contrary to our prediction, a contradiction was found in the mediating effect of work alienation between these two studies. There are several plausible reasons for this unexpected finding. First, the mediating effect of work alienation was not as robust as expected. This may be because the mediators were not randomized during the experiment. Thus, there may be some unobserved post-treatment confounders that could violate the effect of work alienation on knowledge hiding in Study 1 [70], apart from the social contextual factor (TMX) and personal disposition (LGO). Furthermore, job embeddedness is a behavioral-based concept, and its characteristics are much easier to capture and understand. However, work alienation is a psychologically oriented concept, and there is no agreed-upon definition up to now [40]. Thus, we infer that students may be confused in understanding the true value of work alienation compared to job embeddedness, which leads to a less accurate assessment of the perceived level of work alienation [40].

## 7 Theoretical contributions

This study presents two theoretical advances pertaining to the relationship between TMX and knowledge hiding. First, it enhances the understanding of TMX as an important interfering element in inhibiting employees' knowledge hiding behaviors through the lens of SIP [8]. Most extant literature on TMX is developed on the theory of social exchange, which emphasizes the exchange process of psychological or social resources in terms of costs and benefits, especially in the field of knowledge hiding [18]. This study integrates the TMX framework into the SIP theory to distinguish different cognitive processes in which individuals evaluate and interpret workplace affairs under the influence of TMX. Employees play different roles in team social networks and behave distinctively in analyzing and interpreting informational cues acquired from social interactions based on their role sets. Even for certain information, different roles have different emphases, meaning that the direct mechanism of TMX in knowledge hiding is not singular. Rather than just focusing on one influencing mechanism at a time in previous literature, this study rationalizes the interpersonal effects of TMX on individuals' adjustments of their attitudes and behaviors through the role-sending, attention-shifting, and role-modeling mechanisms [8,28], which offers a more comprehensive picture to explore all the influential pathways of TMX on knowledge hiding. Second, extant research in the realm of knowledge hiding mainly focuses on dyadic relationships or interactions, such as leader-member exchange (LMX) [75]. Although extant LMX research elaborates on the accurate underlying mechanisms of how leadership impacts individuals’ intentions to hide knowledge, our increasing interest still calls for a further understanding of the role that horizontal exchange relationships among team members may play in predicting knowledge hiding behaviors. The investigation conducted on TMX has the potential to provide further insights into the unresolved elements left by LMX when scholars attempt to comprehensively rationalize the impact of social exchange relationships on employees' tendencies to withhold knowledge.

The underlying explanatory mechanisms of job embeddedness and work alienation, regarding the relationship between TMX and knowledge hiding, were then examined through the SIP theory. This study responds to Tan et al.’s [18] appeal for more potential intermediary mechanisms through which TMX could influence employees' behavioral choices of knowledge hiding. To the best of our knowledge, this study is the initial investigation into the role of job embeddedness in the context of knowledge hiding and provides a preliminary theoretical basis for the intermediary effect of job embeddedness on the relationship between TMX and knowledge hiding. The extant literature exploring the mediation effect of job embeddedness has exclusively focused on knowledge sharing rather than knowledge hiding [36]. To fill this gap, this study intended to examine and rationalize the motivational process of why employees refuse to hide knowledge in the face of TMX. As an information source, TMX encourages employees to become more embedded in their organization by highlighting the external coworker support and internal psychological attachment gained from social interactions within the workgroup, providing a more accurate understanding of how to reduce knowledge hiding behaviors in teamwork. This research opens the “Black Box” by emphasizing typical characteristics of high-embedded employees, such as greater person-job fit, wider social connections within the organization, and rational estimation for turnover costs [32], which could inhibit an eagerness to engage in knowledge hiding behaviors. In addition, field Study 1 found that employees were less likely to withhold valuable knowledge if they were less alienated from their current job through positive cognitive processing procedures of information acquired from TMX, although this result was not reproduced in experimental Study 2. This is in line with previous literature showing that work alienation could play an intermediary role in transferring the influence of positive social contexts to knowledge hiding [46]. By shedding light on the mediating effects of job embeddedness and work alienation, this study provides more comprehensive explanations and novel perspectives to rationalize the specific way in which TMX could interfere with the emergence of knowledge hiding behaviors.

Finally, this study also responds to the question of when does TMX affect knowledge hiding via job embeddedness and work alienation by integrating LGO into the research model, which is an emerging yet limited topic in the literature. Specifically, we suggest that individuals' perceptions of LGO greatly impact the way in which a social exchange relationship should be treated and the extent to which it would be valued in that environment, which may, in turn, influence individuals' working status and their subsequent knowledge-related behaviors. With the desire to achieve personal development and career advancement, learning-oriented individuals cherish the positive effects of reciprocal social exchanges among team members on developing the best performance zones (more embeddedness or less alienation), which reinforces individuals' enthusiasm to attend team learning or team cooperation activities, such as information sharing [76]. By verifying the moderating role of LGO, this research highlights the boundary conditions under which individuals’ subjective norms can effectively disengage them from knowledge hiding behaviors in team settings. Therefore, this study enriches the literature on goal orientation theory by emphasizing LGO as an imperative personal factor in amplifying the positive effects of TMX in the workplace.

## 8 Practical implications

The rapid development of the knowledge economy has highlighted the importance of knowledge in obtaining organizational competitive advantages and achieving further success. Given that knowledge hiding continues to be widespread in team settings, investing more energy and effort is required in developing harmonious relationships among team members. In this manner, employees can easily gain useful information from the social exchange procedure about what behavior is appreciated and what behavior is prohibited by managers in the teamwork process. Accordingly, managers should consider taking actions, such as encouraging regular performance feedback or team-building activities, to develop stable social networks among team members and strengthen team communication and cooperation. This is also consistent with the tendency of the impact of leadership to wane because of the development of servant leadership and self-managed teams.

This study identified two specific pathways through which TMX could also make a difference in knowledge hiding, which means that the strategy to convert the restraining influence of TMX on knowledge hiding is not unique. Job embeddedness and work alienation have been found to mediate the relationship between TMX and knowledge hiding, indicating that managers should give priority to improve employees' working status as a means to alleviate knowledge hiding behaviors. First, ability-enhancing human resource management (HRM) practices, such as training or personal development, are more effective in embedding employees into their jobs by improving their human capital [77]. In addition, some motivation-enhancing practices, such as higher compensation, rewards, and well-being, could be regarded as employment benefits that could improve employees’ perceptions of job embeddedness [77]. Second, the detrimental effects of work alienation on employee well-being and work outcomes should be emphasized and duly handled by managers. Specifically, employees should be actively involved in the decision-making process to improve their involvement in their daily work. Managers could also empower employees with more control and autonomy to increase their flexibility and discretion in work tasks. The abovementioned practices could dramatically reduce discrepancies between the objective work environment and personal values, making individuals less likely to suffer from work alienation in the workplace.

Finally, this study also provides some implications for LGO in organizations, which could be viewed as a contributory personal disposition to avoid deviant workplace behaviors. Our moderating findings indicate the need to highlight the importance of individual differences in regulating employee work attitudes and behaviors, except for TMX. LGO reflects an individual's intention to achieve further personal development by acquiring new knowledge, skills, and competencies; thus, managers should consider seeking individuals with higher LGO and regard it as an important employment criterion in the hiring process.

## 9 Limitations and further research directions

This study has some limitations. The first limitation concerns the shortage of TMX manipulation. Stable social relationships in teams are usually based on shared perceptions or goals that have developed over time, such as LMX. Therefore, the complex structure of a team's social network cannot be fully reflected through a simple textual narrative [78]. Experimental study 2 adopted hypothetical TMX scenarios as the basis for measuring the proposed relationships with knowledge hiding. This method might have resulted in potential bias because participants could not capture the full extent of TMX relationships, although a similar research protocol for experiments has already been implemented in the field of knowledge hiding [63,79]. However, even though the experimental methodology has shortcomings, it undoubtably compensates for the lack of causal inferences in field studies without longitudinal data in which people could be assessed at more than one point of time [80]. In this case, we suggest that further research could improve our research methodology by conducting field experiments to validate the proposed hypotheses, which would strengthen the internal-external validity and causality of our findings.

Second, we lack a clear interpretation and comprehension regarding the relative significance of LMX and TMX inside the realm of knowledge hiding. The importance of LMX in inhibiting employees’ engagement in knowledge hiding activities has been highlighted in the literature [75,78]. As teams have become the fundamental operational entity within organizations, extant empirical research has already extended the scope of social exchange contexts by highlighting the existence of TMX. However, the existing research exploring the influence of LMX and TMX on knowledge hiding is parallel and independent, and both have been found to exert unique effects on knowledge hiding through different mechanisms. LMX and TMX can simultaneously co-exist in the workplace owing to their similar derivations. However, to this point, Banks et al. [81] suggest that individuals who perceive the significance of high-quality relationships in work settings and want to strengthen such social networks are more likely to exhibit similar attitudes towards both leaders and team members. In this case, this research calls for further investigation of the magnitude of the relative importance of TMX to LMX in explaining the total variance in knowledge hiding.

Third, we need to pay attention to the negative correlation between job embeddedness and work alienation in the framework proposed in Study 1. In similar models with multiple mediators, mediating variables are easily associated because they are influenced by common independent variables. Although serious multicollinearity problems were not found when these two mediators were regressed simultaneously by detecting variance inflation factors (VIF), the significance of the indirect effects, especially the effects of mediators on outcomes, may still be suppressed to the degree to which these mediators are associated with each other [82]. In this case, we also tested the indirect effects of job embeddedness and work alienation separately, and the mediation estimates were slightly higher than those tested together, but still significant in the hypothesized direction. Thus, we suggest that the indirect effects are not seriously affected by intercorrelations between mediators and that the research findings seem to be robust. To ensure the generalizability and reliability of our findings, advanced data analysis techniques, such as Bayesian estimation under the structural equation modeling framework; this could minimize potential bias and standard errors arising from multiple correlated mediators compared to traditional tools.

Finally, the samples of Studies 1 and 2 were from China, and we need to reconsider the generalizability of our research findings to non-Eastern cultures. Scholars inside the realm of knowledge management have emphasized the effect of cultural differences on knowledge hiding. However, relevant research is still lacking. Among the few exceptions, the extant research has focused on individual-related cultural elements rather than on national cultures from different regions, such as cultural intelligence [83] and collectivistic and individualistic values [84]. The importance of indigenous cultures, such as the “guanxi” culture in China, and religious culture, such as Islam in the Middle East or Christianity in the West, should be highlighted in further studies, aiming to better investigate the cultural-conditioning characteristic of knowledge hiding.

## Funding statement

The first author was supported by [blind for the review].

## Data availability statement

The data presented in this study will be openly available at the Mendeley Data: 10.17632/kz8m6g5vmd.1.

## Ethical statement

No animal studies are presented in this manuscript. This study involving human participants was reviewed and approved by the Ethics Committee of the Graduate School of Humanities and Social Sciences, Hiroshima University, Hiroshima, Japan. The participants have confirmed the electronic informed consent to participate in this study.

## CRediT authorship contribution statement

**Zijun Zhang:** Writing – original draft, Visualization, Validation, Software, Resources, Project administration, Methodology, Investigation, Formal analysis, Data curation, Conceptualization. **Yoshi Takahashi:** Writing – review & editing, Validation, Supervision, Methodology, Funding acquisition, Conceptualization.

## Declaration of competing interest

The authors declare that they have no known competing financial interests or personal relationships that could have appeared to influence the work reported in this paper.

## Figures and Tables

**Table:** 

			Levene's Test of Homogeneity of variance		Independent-sample *t*-test	
Variables	Response	Cases	F value	Sig.	T value	Sig. (2-tailed)
Gender	Early Late	114 114	0.876	0.350	0.661	0.510
Age	Early Late	114 114	0.885	0.348	−0.190	0.849
Education	Early Late	114 114	1.322	0.252	−1.060	0.290
Tenure	Early Late	114 114	0.016	0.900	−0.056	0.955
TMX	Early Late	114 114	0.198	0.657	0.508	0.612
LGO	Early Late	114 114	0.011	0.918	0.272	0.786
JE	Early Late	114 114	0.592	0.431	0.667	0.605
WA	Early Late	114 114	0.517	0.473	0.556	0.507
KH	Early Late	114 114	0.028	0.868	0.156	0.876

Notes: TMX: team-member exchange; LGO: learning goal orientation; JE: job embeddedness; WA: work alienation; KH: knowledge hiding.

**Table:** 

			Levene's Test of Homogeneity of variance		Independent-sample *t*-test	
Variables	Response	Cases	F value	Sig.	T value	Sig. (2-tailed)
Gender	Early Late	32 32	0.261	0.611	0.257	0.798
Age	Early Late	32 32	0.466	0.498	0.727	0.470
Education	Early Late	32 32	0.505	0.480	0.354	0.724
Tenure	Early Late	32 32	2.207	0.142	−0.760	0.450
TMX	Early Late	32 32	1.049	0.310	0.737	0.464
LGO	Early Late	32 32	0.280	0.598	−0.404	0.687
JE	Early Late	32 32	0.056	0.813	−0.520	0.605
WA	Early Late	32 32	1.893	0.174	0.668	0.507
KH	Early Late	32 32	0.387	0.536	0.704	0.484

Notes: TMX: team-member exchange; LGO: learning goal orientation; JE: job embeddedness; WA: work alienation; KH: knowledge hiding.
